# Venous-phase Tan-based collateral assessment on CTP-derived CTA for outcome stratification after reperfusion therapy in acute ischemic stroke

**DOI:** 10.3389/fneur.2026.1908458

**Published:** 2026-08-24

**Authors:** Wei Zheng, Huiran Xu, Baoteng Zhang, Li Fu, Kaihe Lin, Suying Wu, Jianfang Huang, Lanying Zhu, Chongchong Dai, Xiaoyan Yu

**Affiliations:** 1 Department of Radiology, The First Hospital of Putian City, Putian, Fujian, China; 2 Department of Neurology, The First Hospital of Putian City, Putian, Fujian, China

**Keywords:** acute ischemic stroke, collateral circulation, computed tomography perfusion, computed tomography angiography, functional outcome, Tan score

## Abstract

**Background:**

Collateral status is closely related to functional outcome after reperfusion therapy in acute ischemic stroke. Conventional single-phase computed tomography angiography (CTA) Tan score may be affected by scan timing and delayed collateral filling, whereas CTA reconstructed from computed tomography perfusion (CTP) source images provides time-resolved vascular information. This study compared conventional CTA Tan score with time-specific Tan-based collateral scores on CTP-derived CTA for predicting 90-day functional outcome.

**Methods:**

This single-center retrospective study included 104 stroke events from 103 patients with acute anterior circulation ischemic stroke who underwent intravenous thrombolysis and/or mechanical thrombectomy. Conventional CTA Tan score and CTP-derived CTA arterial-phase, arteriovenous-phase, and venous-phase time-specific Tan-based scores were assessed. The primary outcome was favorable functional outcome, defined as a modified Rankin Scale score of 0–2 at 90 days. Logistic regression, receiver operating characteristic curve analysis, DeLong tests, treatment-stratified analyses, and exploratory thrombectomy sensitivity analyses were performed.

**Results:**

Sixty-two stroke events were associated with a favorable outcome. Forty-three events were treated with mechanical thrombectomy (37 direct thrombectomy and six bridging therapy), and 61 were treated with intravenous thrombolysis without subsequent thrombectomy. The areas under the curve for conventional CTA Tan score and CTP-derived arterial-phase, arteriovenous-phase, and venous-phase scores were 0.811, 0.811, 0.814, and 0.856, respectively. The venous-phase score showed the highest discriminative performance, although the overall difference compared with conventional CTA Tan score did not reach statistical significance (*p* = 0.079). In multivariable analysis, the venous-phase score remained associated with favorable outcome after adjustment for measured covariates. In the mechanical thrombectomy subgroup, the venous-phase score showed a higher AUC than conventional CTA Tan score (0.833 vs. 0.684; *p* = 0.004), whereas no significant difference was observed in the intravenous thrombolysis subgroup; however, the treatment-by-score interaction was not statistically significant (*p* = 0.788).

**Conclusion:**

The venous-phase Tan-based score on CTP-derived CTA may reflect delayed collateral filling and was associated with 90-day favorable outcome. Its AUC was numerically higher than that of the conventional CTA Tan score, but the difference was not statistically significant; incremental prognostic value remains to be confirmed. The mechanical thrombectomy subgroup finding should be considered exploratory and hypothesis-generating.

## 1 Introduction

Acute ischemic stroke (AIS) is a leading cause of death and disability worldwide (1). Early reperfusion therapy is central to improving functional outcomes (2). Advances in intravenous thrombolysis and mechanical thrombectomy have substantially improved outcomes in patients with acute anterior circulation large-vessel occlusion (3, 4). Multiple randomized controlled trials have shown that mechanical thrombectomy significantly increases functional independence in eligible patients (5). Endovascular treatment also provides clear benefits in patients presenting in the late time window when selected using clinical–imaging mismatch or perfusion imaging criteria (6, 7). Similarly, patients selected based on computed tomography angiography (CTA) collateral status may benefit from endovascular treatment in the late time window (8). Therefore, accurate pretreatment assessment of ischemic tissue status and prognosis remains an important issue in acute stroke imaging.

Collateral circulation is a major determinant of tissue fate and treatment benefit in AIS. Robust leptomeningeal collaterals may delay infarct core expansion, preserve perfusion of the ischemic penumbra, and are associated with better responses to reperfusion therapy and improved functional outcomes (9). Previous studies have shown that CTA-based collateral scores can predict clinical outcomes in patients with AIS. Analyses from the Highly Effective Reperfusion Using Multiple Endovascular Devices (HERMES) collaboration and other studies further demonstrated associations between different CTA collateral scores and functional outcomes after endovascular treatment (10). The Tan collateral score is a simple and intuitive CTA-based method and has been widely used to assess collateral circulation in acute anterior circulation ischemic stroke (11). However, conventional single-phase CTA has inherent limitations. It captures only a single time point during contrast passage and may be affected by scan timing, cardiac output, the degree of proximal occlusion, and delayed collateral filling (12). Consequently, conventional CTA may underestimate collateral status in patients with delayed collateral filling (13). By contrast, multiphase CTA, dynamic CTA, and CTA reconstructed from computed tomography perfusion (CTP) source images can visualize contrast passage from the arterial to the venous phase (14), thereby providing a more comprehensive assessment of the temporal characteristics of collateral filling. Previous studies have suggested that these dynamic or multiphase imaging approaches may improve the reliability of collateral assessment (15, 16).

In clinical practice, CTP is widely used to evaluate patients with AIS. In addition to conventional perfusion parameters, CTP source images contain time-resolved vascular enhancement information that can be used to reconstruct CTA images at different phases (13). Theoretically, venous-phase CTA reconstructed from CTP source images may better depict delayed leptomeningeal collateral filling (17, 18), thereby addressing limitations of conventional single-phase CTA (19). However, few studies have applied the Tan scoring criteria to different phases of CTP-derived CTA and directly compared their prognostic value with conventional CTA Tan scores after reperfusion therapy. Therefore, this study compared the ability of the conventional CTA Tan score and arterial-, arteriovenous-, and venous-phase time-specific collateral scores based on the Tan criteria on CTP-derived CTA to discriminate 90-day functional outcomes in patients with acute anterior circulation ischemic stroke undergoing reperfusion therapy. We further examined whether their performance differed between patients treated with mechanical thrombectomy and those treated with intravenous thrombolysis. We hypothesized that the venous-phase time-specific Tan-based score on CTP-derived CTA would capture delayed collateral filling beyond that shown by conventional single-phase CTA and improve prognostic stratification after reperfusion therapy.

## 2 Materials and methods

### 2.1 Study design and participants

**Figure 1:**
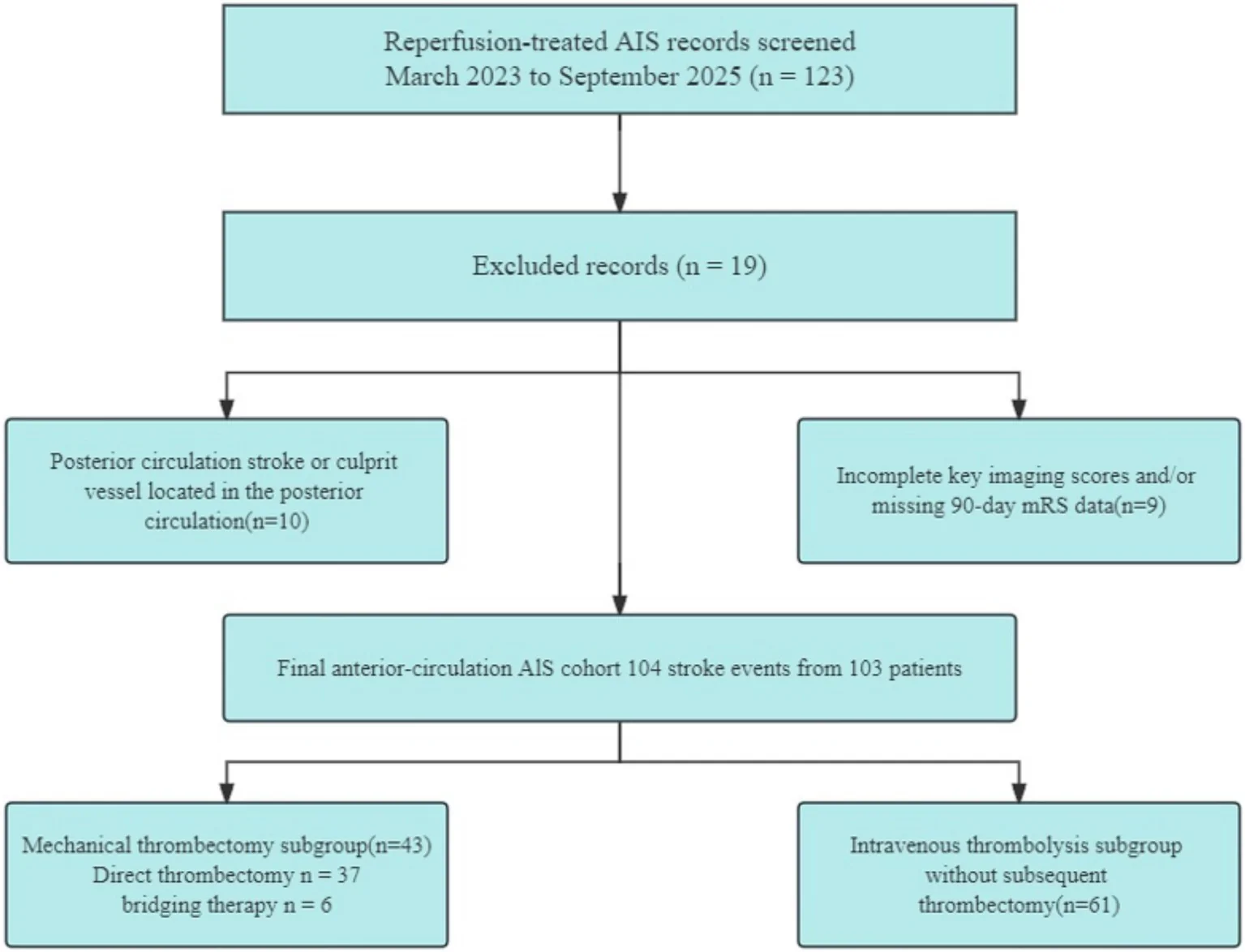
Patient-selection flowchart. A total of 123 reperfusion-treated AIS records were screened; 19 were excluded, and 104 anterior-circulation stroke events from 103 patients were included in the final analysis.

This single-center retrospective cohort study was approved by the Ethics Committee of the First Hospital of Putian City (approval No. 2025–110), which waived the requirement for informed consent. We screened reperfusion-treated AIS records consecutively from the institutional stroke imaging and clinical database at the First Hospital of Putian City between March 2023 and September 2025, and all data were anonymized before analysis. At our center, non-contrast computed tomography (CT), conventional CTA, and CTP are routinely included in the emergency imaging protocol for reperfusion candidates when clinically feasible. Because the Tan collateral score was developed primarily for anterior circulation collateral assessment, only patients with acute anterior circulation ischemic stroke were included. Patients with posterior circulation stroke or a culprit vessel in the posterior circulation were excluded. The inclusion criteria were: ① a clinical diagnosis of AIS; ② treatment with intravenous thrombolysis and/or mechanical thrombectomy; ③ availability of pretreatment non-contrast CT, conventional CTA, and CTP; ④ availability of CTP source images suitable for reconstructing multiphase CTA; and ⑤ available 90-day modified Rankin Scale (mRS) data. The exclusion criteria were: ① posterior circulation stroke or posterior circulation culprit vessel occlusion; ② insufficient image quality for collateral assessment; ③ incomplete baseline imaging data; and ④ unavailable 90-day functional outcome data. Duplicate patient identifiers were reviewed individually. One patient experienced two temporally distinct stroke events, and both events were retained as separate treatment episodes for analysis. The cohort derivation process is shown in Figure 1. The mechanical thrombectomy group included direct mechanical thrombectomy and bridging intravenous thrombolysis followed by mechanical thrombectomy. Functional outcome was classified according to the 90-day mRS score: an mRS score of 0–2 was defined as a favorable outcome, whereas an mRS score of 3–6 was defined as an unfavorable outcome.

### 2.2 Image acquisition and reconstruction of CTA from CTP source images

All CTA and CTP examinations were performed using a dual-source CT scanner (SOMATOM Force; Siemens Healthineers, Erlangen, Germany). Non-contrast head CT was first performed from the skull base to the vertex using the following parameters: tube voltage, 100 kV; automatic tube current modulation (CARE Dose 4D); slice thickness, 5 mm; slice interval, 5 mm; reconstruction thickness, 1 mm; rotation time, 1 s; and collimation, 128 × 0.6 mm. Whole-brain dynamic CTP was subsequently performed from the skull base to the vertex. The acquisition parameters were as follows: tube voltage, 70 kV; effective tube current-time product, 200 mAs; slice thickness, 5 mm; slice interval, 3 mm; reconstruction thickness, 1 mm; rotation time, 0.5 s; detector coverage, 176 mm; and 30 acquisitions at 1.5-s intervals, with a total acquisition time of 45 s. Scanning was initiated simultaneously with contrast injection, and image acquisition began after a 5-s delay. Conventional CTA was performed 8–10 min after completion of CTP to reduce residual contrast contamination from the preceding CTP acquisition and improve the interpretability of subsequent CTA images. No patient received intravenous thrombolysis during the interval between completion of CTP and acquisition of conventional CTA. Dual-energy CTA covered the region from the aortic arch to the vertex. A bolus-tracking technique was used, and scanning commenced automatically 4 s after attenuation in the aortic arch exceeded 100 Hounsfield units (HU). The acquisition parameters were as follows: tube voltages, 80 kV and Sn150 kV; automatic tube current modulation (CARE Dose 4D); collimation, 128 × 0.6 mm; rotation time, 0.25 s; pitch, 0.7; slice thickness, 4 mm; slice interval, 4 mm; and reconstruction thickness, 1.0 mm. For both CTP and CTA, 50 mL of iodinated contrast medium (iopromide, 370 mg I/mL) was administered through an antecubital vein using a dual-head power injector at 4.5 mL/s, followed by 40 mL of saline at the same injection rate. Images were reconstructed using advanced modeled iterative reconstruction (ADMIRE). The whole-brain CTP and CTA datasets were transferred to a Siemens workstation (syngo.via; Siemens Healthineers) for post-processing and image analysis. Time–attenuation curves (TACs) were obtained by manually placing regions of interest (ROIs). An arterial input ROI was placed in the contralateral internal carotid artery, and the arterial enhancement curve was generated using automatic detection. A venous ROI was placed in the confluence of sinuses to generate the venous enhancement curve. Three representative time points were selected from the arterial input and venous output curves to reconstruct CTA images. The arterial phase was defined as the time point near the peak of the arterial input curve. The arteriovenous phase was selected after the arterial peak, when the descending arterial curve approached or intersected the ascending venous curve. The venous phase was defined as the time point near the peak of the venous output curve. To ensure consistent phase definitions, markedly delayed washout-phase images acquired after the venous peak were not included in venous-phase scoring. These reconstructed images were used as the arterial-, arteriovenous-, and venous-phase CTP-derived CTA datasets for subsequent collateral assessment.

### 2.3 Tan collateral scoring and time-specific collateral assessment on CTP-derived CTA

**Figure 2:**
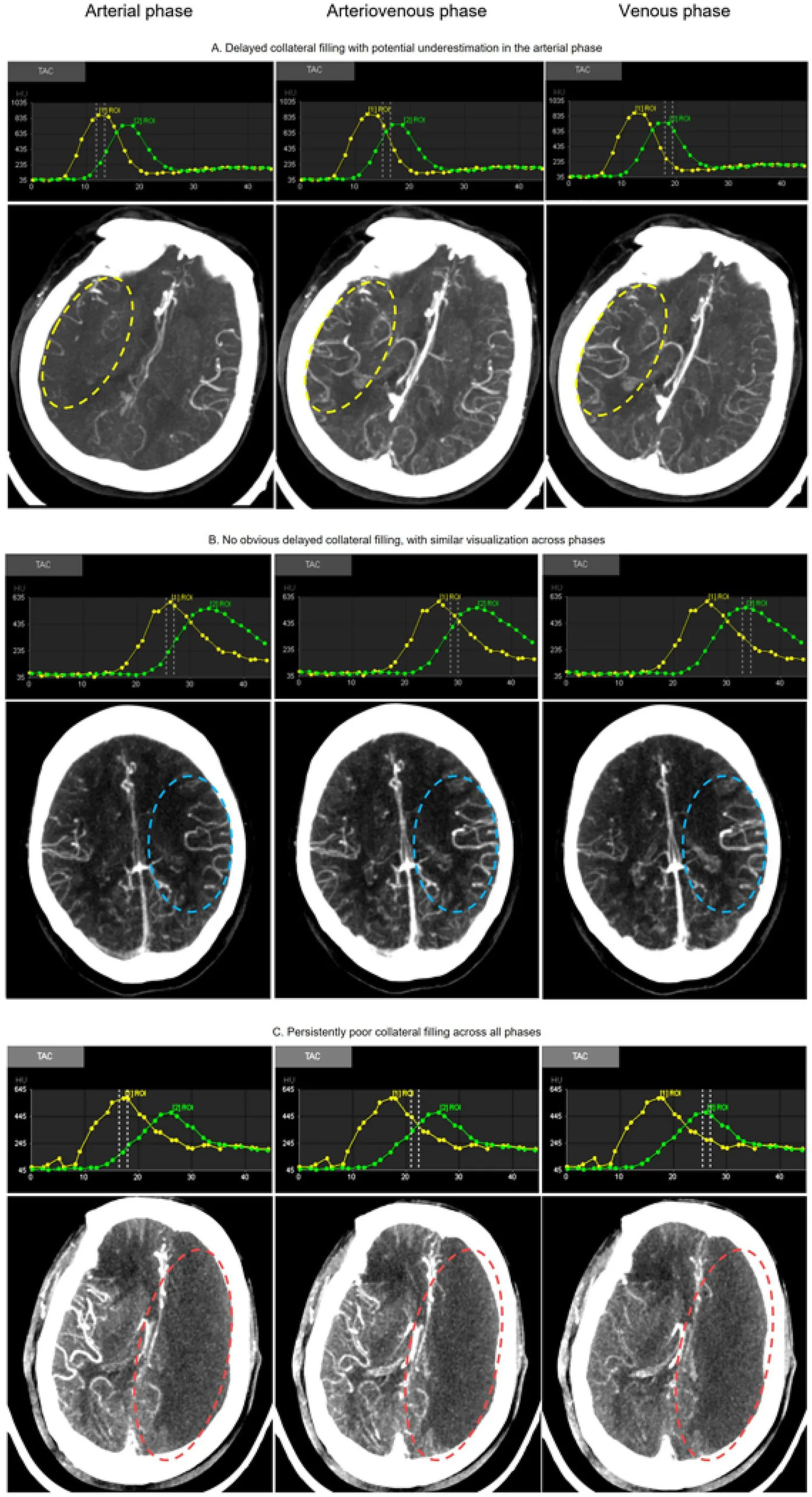
Representative patterns of time-specific Tan-based collateral assessment on CTP-derived CTA. The yellow curve represents the arterial ROI time-attenuation curve, and the green curve represents the venous ROI time-attenuation curve. CTA images were reconstructed at the arterial, arteriovenous, and venous phases according to the selected TAC time points. Row **(A)** shows delayed collateral filling, in which collateral vessels are poorly visualized in the arterial phase but become more evident in later phases, suggesting potential underestimation by early arterial-phase or single-phase assessment. Row **(B)** shows no obvious delayed collateral filling, with similar collateral visualization across phases. Row **(C)** shows persistently poor collateral filling across all phases, indicating that venous-phase assessment does not artificially upgrade the collateral score when effective collateral filling is absent. CTP, computed tomography perfusion; CTA, computed tomography angiography; TAC, time-attenuation curve; ROI, region of interest.

Four collateral scores were recorded for each stroke event: (1) the conventional CTA Tan score (cCTA-Tan); (2) the arterial-phase CTP-derived CTA Tan-based score (CTP-A-Tan); (3) the arteriovenous-phase CTP-derived CTA Tan-based score (CTP-AV-Tan); and (4) the venous-phase CTP-derived CTA Tan-based score (CTP-V-Tan). All scores ranged from 0 to 3, with higher scores indicating better collateral circulation (9, 11). The cCTA-Tan was assessed using pretreatment conventional CTA images. Because the Tan score was originally developed for collateral assessment on single-phase CTA, its criteria were applied to the multiphase CTA images reconstructed from CTP source images, primarily according to the extent of leptomeningeal collateral filling within the vascular territory distal to the occlusion. Therefore, the scores obtained from the different phases of CTP-derived CTA were methodologically defined as time-specific Tan-based collateral scores rather than conventional Tan scores. CTP-V-Tan assessment was restricted to images acquired near the peak of the venous output curve and was used to evaluate delayed arterial-like collateral filling within the affected vascular territory. Persistent enhancement or delayed washout after the venous peak was not considered when assigning the CTP-V-Tan score (Figure 2). Before assessing CTP-V-Tan, the readers reviewed conventional CTA and the arterial and arteriovenous-phase CTP-derived CTA images to identify the culprit vessel, occlusion site, and anatomical extent of the affected vascular territory. Therefore, venous-phase scoring was blinded to clinical outcome but was not fully blinded to preceding imaging phases. During venous-phase assessment, only delayed enhancing vessels within the affected territory that followed the expected course of leptomeningeal collaterals or distal arteries were included. Prominent cortical draining veins, venous sinuses, and large draining veins were excluded from the assessment. When arterial-like collateral vessels could not be reliably distinguished from venous structures on maximum-intensity projection images, thin-section source images, consecutive slices, and multiplanar reconstructions were reviewed. If uncertainty remained, the two readers reached a consensus, with care taken to avoid misclassifying typical venous enhancement as collateral filling. All images were independently assessed by two senior stroke imaging radiologists using a Siemens workstation. Disagreements were resolved through discussion with a third radiologist with greater experience in stroke imaging. Interobserver agreement was evaluated using the quadratic-weighted kappa coefficient, together with the exact agreement rate. For additional clinical interpretation, CTP-V-Tan was dichotomized as 3 versus 0–2. This threshold was clinically motivated rather than selected by ROC cut-off optimization. A Tan grade of 3 represents complete collateral filling of the affected territory, whereas grades 0–2 indicate incomplete filling to varying degrees. Because venous-phase images allow delayed collateral filling to become visible, a score of 2 was still considered incomplete rather than unequivocally good collateralization. The Tan grading criteria were defined as follows: 0, absent collateral filling in the occluded vascular territory; 1, collateral filling of 50% or less of the territory; 2, collateral filling of more than 50% but less than 100% of the territory; and 3, complete collateral filling of the territory. The two primary readers each had more than 10 years of experience in stroke imaging, and the third adjudicating radiologist had more than 15 years of experience. The readers were blinded to the 90-day functional outcomes. Interobserver agreement was calculated from the initial independent ratings obtained before consensus adjudication. For distal Middle Cerebral Artery (MCA), Anterior Cerebral Artery (ACA), or non-large-vessel-occlusion presentations, Tan-based grading was applied to the clinically and radiologically affected anterior-circulation territory rather than assumed to represent the entire MCA territory. Because this application is less standardized than proximal Internal Carotid Artery **(**ICA) or MCA occlusion assessment, additional sensitivity analyses restricted to ICA/M1 and ICA/M1/M2 involvement were performed.

### 2.4 Clinical and imaging variables

The collected clinical variables included age, sex, hypertension, diabetes mellitus, atrial fibrillation, treatment modality, and baseline National Institutes of Health Stroke Scale (NIHSS) score. Treatment modality was categorized as mechanical thrombectomy or intravenous thrombolysis. Imaging variables included the Alberta Stroke Program Early CT Score (ASPECTS) on the affected side, cCTA-Tan, CTP-A-Tan, CTP-AV-Tan, and CTP-V-Tan. ASPECTS was recorded for the affected hemisphere. The affected side was verified according to the documented culprit vessel, occlusion site, and imaging findings. For bilateral anterior circulation lesions, the lower ASPECTS was used for analysis. However, ASPECTS is anatomically less specific in ACA-territory, distal-branch, or non-large-vessel-occlusion presentations than in ICA/MCA-territory ischemia. For thrombectomy-treated events, the modified Thrombolysis in Cerebral Infarction (mTICI) grade, onset-to-recanalization time, infarct core volume, and hypoperfusion volume were extracted from procedural and CTP records for exploratory sensitivity analyses. Although CTP maps were generated in clinical practice, standardized patient-level Cerebral Blood Flow (CBF), Cerebral Blood Volume (CBV), and Mean Transit Time (MTT) values were not consistently recorded across the retrospective cohort in a form suitable for analysis. Therefore, the primary analysis focused on collateral scores derived from CTP source-image reconstructions. In the thrombectomy subgroup, infarct core volume and hypoperfusion volume were available and were explored in sensitivity analyses.

### 2.5 Outcome definitions

The primary outcome was functional status at 90 days. A modified Rankin Scale (mRS) score of 0–2 was defined as a favorable functional outcome, whereas a score of 3–6 was defined as an unfavorable functional outcome. Patients with confirmed death, including those who died before completion of the 90-day follow-up assessment, were assigned an mRS score of 6. Radiological hemorrhagic transformation within 48 h after treatment was recorded as a descriptive post-treatment imaging finding. Hemorrhagic transformation was assessed according to the European Cooperative Acute Stroke Study I (ECASS I) imaging criteria and recorded as a binary variable. Hemorrhagic infarction type 1 or 2 and parenchymal hematoma type 1 or 2 were classified as hemorrhagic transformation (20). The ECASS I criteria were used because hemorrhagic transformation had been retrospectively recorded according to ECASS I categories, and these data were consistently retrievable from the imaging records. The discriminative performance of the collateral scores for 90-day favorable functional outcome was additionally explored in the mechanical thrombectomy and intravenous thrombolysis subgroups.

### 2.6 Statistical analysis

Continuous variables are presented as mean ± standard deviation or median with interquartile range, and categorical variables as counts and percentages. Distributional assumptions were assessed using histograms and quantile-quantile plots. Age was approximately symmetric on graphical assessment and was compared between outcome groups using Welch’s two-sample t-test. Ordinal or skewed continuous variables, including NIHSS, ASPECTS, and collateral scores, were compared using the Wilcoxon rank-sum test. Categorical variables were compared using Fisher’s exact test. Missing data were not imputed. Analyses were performed using available data, and multivariable models used complete-case analysis. Baseline NIHSS and affected-side ASPECTS were each unavailable for one different stroke event; therefore, 102 events were included in the multivariable models. Data on hemorrhagic transformation within 48 h were unavailable for nine events because follow-up imaging within this time window was not available. Univariable logistic regression was used to assess the associations of baseline clinical and imaging variables with favorable functional outcome at 90 days. Multivariable logistic regression was subsequently performed to determine whether CTP-V-Tan was associated with favorable outcome after adjustment for measured covariates. Age, baseline NIHSS score, affected-side ASPECTS, and treatment modality were selected *a priori* as adjustment variables because they represent clinically relevant demographic, stroke-severity, baseline imaging, and treatment factors. The number of covariates was limited in view of the sample size and number of outcome events. Results are reported as odds ratios (ORs) with 95% confidence intervals (CIs). The following multivariable models were constructed: (1) clinical covariates plus cCTA-Tan; (2) clinical covariates plus CTP-V-Tan; (3) clinical covariates plus both cCTA-Tan and CTP-V-Tan; and (4) clinical covariates plus dichotomized CTP-V-Tan, defined as 3 versus 0–2 as an exploratory clinically motivated threshold. Spearman correlation and variance inflation factors (VIFs) were calculated to assess the relationship and potential multicollinearity between cCTA-Tan and CTP-V-Tan in the combined model. Receiver operating characteristic (ROC) curve analysis was used to evaluate the ability of cCTA-Tan, CTP-A-Tan, CTP-AV-Tan, and CTP-V-Tan to discriminate favorable functional outcome at 90 days. The area under the curve (AUC) was calculated for each score. Differences in AUC between cCTA-Tan and each time-specific CTP-derived CTA score were compared using the DeLong test. Treatment-stratified analyses were performed to explore the difference in discriminative performance between CTP-V-Tan and cCTA-Tan within the mechanical thrombectomy and intravenous thrombolysis subgroups. In the mechanical thrombectomy subgroup, exploratory sensitivity models evaluated CTP-V-Tan after separate adjustment for onset-to-recanalization time, infarct core volume, or hypoperfusion volume. The mTICI grade was summarized descriptively; because all thrombectomy events achieved successful reperfusion, it was not entered as an additional regression covariate. An adjusted interaction model including treatment modality, CTP-V-Tan, and their interaction term was used to assess whether the association between CTP-V-Tan and outcome differed by treatment modality. An exploratory heatmap was generated to visualize 90-day favorable outcome proportions across combinations of cCTA-Tan and CTP-V-Tan scores, particularly within intermediate cCTA-Tan categories. Because several cells contained few observations, the heatmap was interpreted descriptively and was not used for formal hypothesis testing. All final statistical analyses were performed using R version 4.6.0. ROC analyses and DeLong tests were conducted using the pROC package version 1.19.0.1. A two-sided *p* < 0.05 was considered statistically significant. To address the anatomical standardization of Tan-based collateral assessment, ROC analyses were repeated in events with ICA or M1 involvement and in events with ICA, M1, or M2 involvement.

## 3 Results

### 3.1 Baseline characteristics

A total of 123 consecutive reperfusion-treated AIS records were screened. Nineteen records were excluded, including 10 with posterior circulation stroke or a posterior circulation culprit vessel and nine with incomplete key imaging scores and/or missing 90-day mRS data. The final cohort comprised 103 patients with acute anterior circulation ischemic stroke contributing 104 stroke events treated with reperfusion therapy (Figure 1). Of the 104 stroke events, 43 were treated with mechanical thrombectomy and 61 with intravenous thrombolysis without subsequent thrombectomy. The mechanical thrombectomy subgroup included 37 direct thrombectomy events and six bridging therapy events. At 90 days, 62 stroke events were associated with a favorable functional outcome and 42 with an unfavorable outcome. The culprit vessel or occlusion pattern was distributed as follows: MCA M1, 44 events (42.3%); ICA with tandem MCA involvement, 20 events (19.2%); no visible major anterior-circulation occlusion, 15 events (14.4%); MCA M2, 11 events (10.6%); isolated ICA, nine events (8.7%); and ACA, five events (4.8%). Overall, 72 events (69.2%) had ICA or M1 involvement, 84 events (80.8%) had ICA, M1, or M2 involvement, and 89 events (85.6%) had visible anterior-circulation occlusion. Compared with the favorable outcome group, the unfavorable outcome group had a higher prevalence of atrial fibrillation (52.4% vs. 29.0%, *p* = 0.024), higher baseline NIHSS scores (17.5 (12.2–21.0) vs. 10.0 (6.0–14.0), *p* < 0.001), and lower affected-side ASPECTS (8.0 (6.0–8.0) vs. 9.0 (8.0–10.0), *p* < 0.001). Age, sex, hypertension, diabetes mellitus, and treatment modality did not differ significantly between the two groups. Detailed baseline characteristics are presented in Table 1.

**Table 1:** Baseline clinical and imaging characteristics.

Variable	Overall (*n* = 104 events)	Favorable outcome (*n* = 62 events)	Unfavorable outcome (*n* = 42 events)	*P*
Age, years	69.6 ± 13.8	68.4 ± 14.0	71.5 ± 13.3	0.261
Male sex	68/104 (65.4%)	44/62 (71.0%)	24/42 (57.1%)	0.207
Hypertension	65/104 (62.5%)	38/62 (61.3%)	27/42 (64.3%)	0.838
Diabetes mellitus	43/104 (41.3%)	25/62 (40.3%)	18/42 (42.9%)	0.841
Atrial fibrillation	40/104 (38.5%)	18/62 (29.0%)	22/42 (52.4%)	0.024
Mechanical thrombectomy	43/104 (41.3%)	21/62 (33.9%)	22/42 (52.4%)	0.070
Direct thrombectomy	37/104 (35.6%)	18/62 (29.0%)	19/42 (45.2%)	—
Bridging therapy	6/104 (5.8%)	3/62 (4.8%)	3/42 (7.1%)	—
Baseline NIHSS	13.0 (8.0–19.0)	10.0 (6.0–14.0)	17.5 (12.2–21.0)	< 0.001
Affected-side ASPECTS	8.0 (7.0–10.0)	9.0 (8.0–10.0)	8.0 (6.0–8.0)	< 0.001
cCTA-Tan	2.0 (1.0–3.0)	2.5 (2.0–3.0)	1.0 (1.0–2.0)	< 0.001
CTP-A-Tan	2.0 (1.0–3.0)	2.5 (2.0–3.0)	1.0 (1.0–2.0)	< 0.001
CTP-AV-Tan	2.0 (1.0–3.0)	3.0 (2.0–3.0)	1.0 (1.0–2.0)	< 0.001
CTP-V-Tan	3.0 (2.0–3.0)	3.0 (3.0–3.0)	2.0 (1.0–2.0)	< 0.001

Data are presented as mean ± standard deviation, median (interquartile range), or n/*N* (%), as appropriate. Age was compared using Welch’s two-sample *t*-test; ordinal or non-normally distributed variables were compared using the Wilcoxon rank-sum test; and categorical variables were compared using Fisher’s exact test. Direct thrombectomy and bridging therapy are shown as descriptive subcategories of the mechanical thrombectomy group. Baseline NIHSS and affected-side ASPECTS were each available for 103 stroke events. NIHSS, National Institutes of Health Stroke Scale; ASPECTS, Alberta Stroke Program Early CT Score; cCTA-Tan, conventional CTA Tan score; CTP-A-Tan, arterial-phase CTP-derived CTA Tan-based score; CTP-AV-Tan, arteriovenous-phase CTP-derived CTA Tan-based score; CTP-V-Tan, venous-phase CTP-derived CTA Tan-based score.

### 3.2 Interobserver agreement

Interobserver agreement between the two radiologists was good to excellent for all four collateral scores. The exact agreement rates for cCTA-Tan, CTP-A-Tan, CTP-AV-Tan, and CTP-V-Tan were 99.0, 84.6, 97.1, and 84.6%, respectively. The corresponding quadratic-weighted kappa values were 0.993, 0.878, 0.979, and 0.868.

### 3.3 Discriminative performance of the collateral scores for 90-day favorable outcome

**Figure 3:**
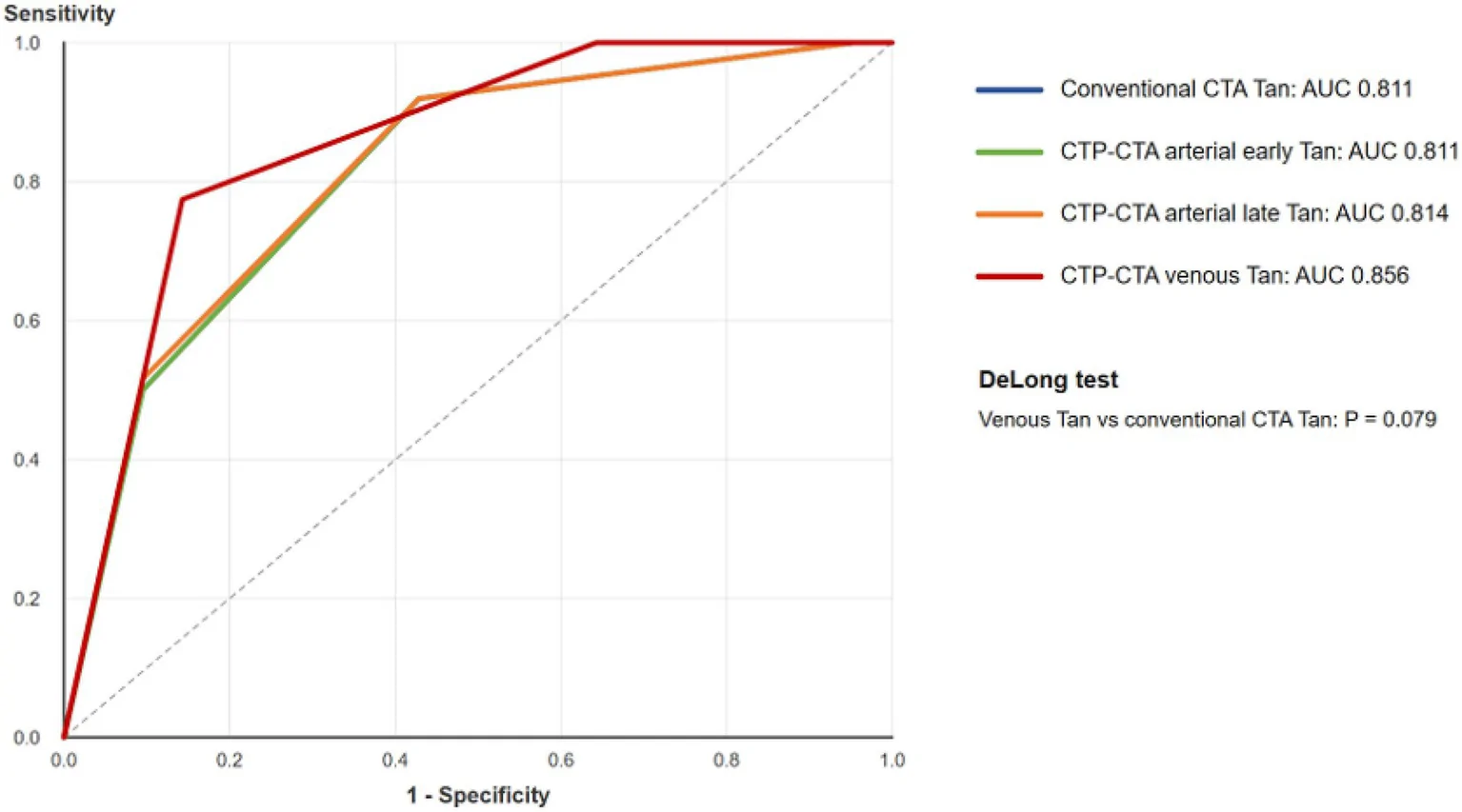
ROC curves of collateral scores for discriminating 90-day favorable functional outcome. The AUCs for cCTA-Tan, CTP-A-Tan, CTP-AV-Tan, and CTP-V-Tan were 0.811, 0.811, 0.814, and 0.856, respectively. The AUC of CTP-V-Tan was numerically higher than that of cCTA-Tan, although the difference was not statistically significant according to the DeLong test (*p* = 0.079).

ROC analysis showed that the AUCs of cCTA-Tan, CTP-A-Tan, CTP-AV-Tan, and CTP-V-Tan for discriminating 90-day favorable functional outcome were 0.811, 0.811, 0.814, and 0.856, respectively. Compared with cCTA-Tan, CTP-V-Tan increased the AUC by 0.045; however, the difference did not reach statistical significance according to the DeLong test (95% CI, −0.005 to 0.096; *p* = 0.079). The ROC curves are shown in Figure 3. In sensitivity analyses restricted to more anatomically standardized occlusion groups, CTP-V-Tan remained numerically higher than cCTA-Tan. Among events with ICA or M1 involvement (*n* = 72), the AUCs were 0.886 for CTP-V-Tan and 0.829 for cCTA-Tan. Among events with ICA, M1, or M2 involvement (*n* = 84), the corresponding AUCs were 0.860 and 0.801, respectively.

### 3.4 Univariable logistic regression analysis

In univariable logistic regression, atrial fibrillation was associated with lower odds of favorable outcome (OR = 0.372; 95% CI, 0.164–0.842; *p* = 0.018). Higher baseline NIHSS scores were also associated with lower odds of favorable outcome (OR per 1-point increase = 0.873; 95% CI, 0.818–0.933; *p* < 0.001), whereas higher affected-side ASPECTS was associated with higher odds of favorable outcome (OR per 1-point increase = 1.806; 95% CI, 1.364–2.391; *p* < 0.001). All four collateral scores were associated with favorable outcome, with the largest univariable effect estimate observed for CTP-V-Tan (OR per 1-point increase = 15.111; 95% CI, 5.672–40.259; *p* < 0.001). Full univariable results are provided in Table 2.

**Table 2:** Univariable logistic regression analyses for 90-day favorable functional outcome.

Variable	*n*	OR	95% CI	*p*-value
Age, per 1-year increase	104	0.983	0.954–1.013	0.265
Male sex	104	1.833	0.807–4.167	0.148
Hypertension	104	0.880	0.390–1.982	0.757
Diabetes mellitus	104	0.901	0.407–1.994	0.797
Atrial fibrillation	104	0.372	0.164–0.842	0.018
Mechanical thrombectomy	104	0.466	0.209–1.038	0.062
Baseline NIHSS, per 1-point increase	103	0.873	0.818–0.933	< 0.001
Affected-side ASPECTS, per 1-point increase	103	1.806	1.364–2.391	< 0.001
cCTA-Tan, per 1-point increase	104	6.118	2.996–12.495	< 0.001
CTP-A-Tan, per 1-point increase	104	6.118	2.996–12.495	< 0.001
CTP-AV-Tan, per 1-point increase	104	6.172	3.031–12.570	< 0.001
CTP-V-Tan, per 1-point increase	104	15.111	5.672–40.259	< 0.001

Odds ratios for continuous and ordinal variables are reported per 1-unit increase. Female sex, absence of hypertension, absence of diabetes mellitus, absence of atrial fibrillation, and intravenous thrombolysis without subsequent thrombectomy served as reference categories. NIHSS, National Institutes of Health Stroke Scale; ASPECTS, Alberta Stroke Program Early CT Score; OR, odds ratio; CI, confidence interval.

### 3.5 Multivariable logistic regression analysis

**Figure 4:**
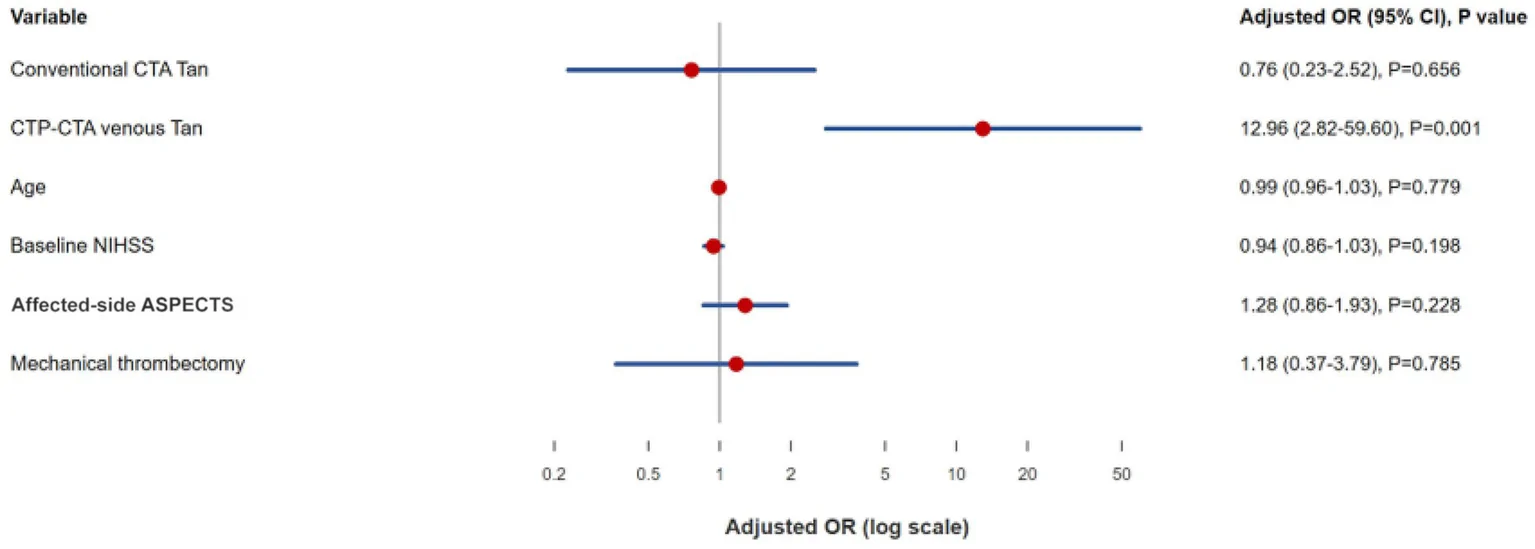
Adjusted odds ratios for 90-day favorable functional outcome in the combined model. The model simultaneously included cCTA-Tan and CTP-V-Tan and was adjusted for age, baseline NIHSS score, affected-side ASPECTS, and treatment modality. CTP-V-Tan remained statistically associated with favorable outcome, whereas cCTA-Tan was not statistically significant. Given the high correlation between the scores and the wide confidence interval for CTP-V-Tan, the individual estimates should be interpreted cautiously and not as evidence of independent effects or statistical superiority. Red circles indicate adjusted odds ratios, and horizontal lines indicate 95% confidence intervals. The vertical reference line represents an odds ratio of 1.

After adjustment for age, baseline NIHSS score, affected-side ASPECTS, and treatment modality, cCTA-Tan was associated with favorable functional outcome at 90 days (OR = 3.684; 95% CI, 1.655–8.200; *p* = 0.001). When CTP-V-Tan was entered into the model instead of cCTA-Tan, CTP-V-Tan was associated with favorable outcome (OR = 10.193; 95% CI, 3.510–29.602; *p* < 0.001). When cCTA-Tan and CTP-V-Tan were simultaneously included in the same multivariable model, the cCTA-Tan estimate changed markedly and was not statistically significant (OR = 0.762; 95% CI, 0.230–2.519; *p* = 0.656), whereas CTP-V-Tan remained statistically associated with favorable outcome, although with a wide confidence interval (OR = 12.957; 95% CI, 2.817–59.603; *p* = 0.001). cCTA-Tan and CTP-V-Tan were highly correlated (Spearman rho = 0.822; *p* < 0.001). The VIFs for cCTA-Tan and CTP-V-Tan were 3.45 and 3.33, respectively. Although these values were below commonly used thresholds for severe multicollinearity, the high correlation, marked coefficient change, and wide confidence interval indicated substantial shared information and limited precision. Accordingly, the individual coefficients should be interpreted cautiously and not as evidence of independent effects or statistical superiority. After dichotomization, a CTP-V-Tan score of 3 was associated with favorable outcome (OR = 11.065; 95% CI, 3.502–34.959; *p* < 0.001). The multivariable logistic regression results are presented in Table 3 and Figure 4.

**Table 3:** Multivariable logistic regression analyses for 90-day favorable functional outcome.

Model	Variable of interest	Adjusted OR	95% CI	*P*
Model 1	cCTA-Tan, per 1-point increase	3.684	1.655–8.200	0.001
Model 2	CTP-V-Tan, per 1-point increase	10.193	3.510–29.602	< 0.001
Model 3	cCTA-Tan, per 1-point increase	0.762	0.230–2.519	0.656
	CTP-V-Tan, per 1-point increase	12.957	2.817–59.603	0.001
Model 4	CTP-V-Tan score of 3 (vs. 0–2)	11.065	3.502–34.959	< 0.001

Model 1 included cCTA-Tan; Model 2 included CTP-V-Tan; Model 3 included both cCTA-Tan and CTP-V-Tan; and Model 4 included dichotomized CTP-V-Tan, with a score of 3 indicating complete delayed collateral filling on venous-phase CTP-derived CTA and scores of 0–2 serving as the reference category. All models were adjusted for age, baseline NIHSS score, affected-side ASPECTS, and treatment modality. Intravenous thrombolysis without subsequent thrombectomy was the reference category for treatment modality. In Models 1–3, adjusted ORs for cCTA-Tan and CTP-V-Tan are reported per 1-point increase in score. OR, odds ratio; CI, confidence interval; NIHSS, National Institutes of Health Stroke Scale; ASPECTS, Alberta Stroke Program Early CT Score; cCTA-Tan, conventional CTA Tan score; CTP-V-Tan, venous-phase CTP-derived CTA Tan-based score.

### 3.6 Treatment subgroup analysis

**Figure 5:**
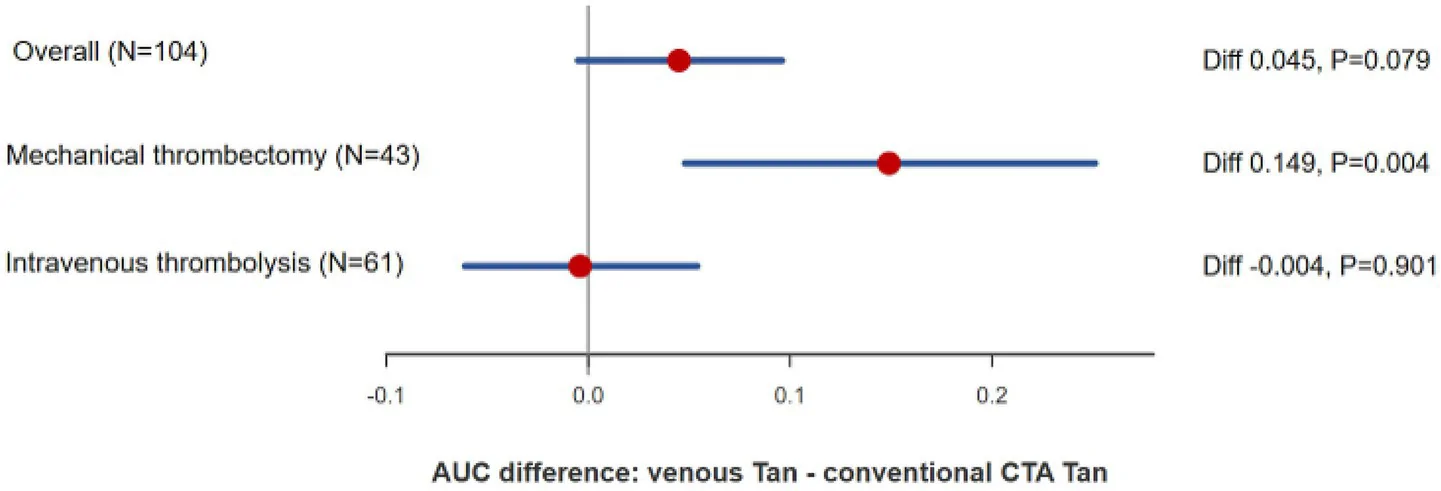
Differences in discriminative performance between CTP-V-Tan and cCTA-Tan according to treatment modality. Points indicate differences in AUC calculated as CTP-V-Tan minus cCTA-Tan, and horizontal lines indicate 95% confidence intervals. CTP-V-Tan showed a higher AUC than cCTA-Tan in the mechanical thrombectomy subgroup (difference, 0.149; 95% CI, 0.048–0.251; *p* = 0.004), whereas no significant difference was observed in the intravenous thrombolysis subgroup. The treatment-by-CTP-V-Tan interaction term was not statistically significant.

In the mechanical thrombectomy subgroup, the AUCs of cCTA-Tan and CTP-V-Tan for discriminating 90-day favorable functional outcome were 0.684 and 0.833, respectively. CTP-V-Tan increased the AUC by 0.149 compared with cCTA-Tan, and the difference was statistically significant according to the DeLong test (95% CI, 0.048–0.251; *p* = 0.004). In the intravenous thrombolysis subgroup, the AUCs of cCTA-Tan and CTP-V-Tan were 0.856 and 0.852, respectively, with no significant difference between the two scores (AUC difference, −0.004; 95% CI, −0.061 to 0.054; *p* = 0.901). The treatment-by-CTP-V-Tan interaction term was not statistically significant in the adjusted interaction model (*p* = 0.788); therefore, the treatment-stratified findings should be interpreted as exploratory rather than definitive evidence that relative score performance differs by treatment modality. All 43 thrombectomy events achieved successful reperfusion (mTICI 2b, *n* = 14; mTICI 3, *n* = 29). In exploratory thrombectomy sensitivity analyses, CTP-V-Tan remained associated with favorable outcome after adjustment for onset-to-recanalization time (OR = 14.78; 95% CI, 2.77–78.89; *p* = 0.002), and in separate models additionally considering infarct core volume (OR = 15.50; 95% CI, 2.74–87.53; *p* = 0.002) or hypoperfusion volume (OR = 15.11; 95% CI, 2.67–85.67; *p* = 0.002). The subgroup ROC comparisons are presented in Table 4 and Figure 5.

**Table 4:** Comparison of cCTA-Tan and CTP-V-Tan for discriminating 90-day favorable functional outcome.

Subgroup	cCTA-Tan AUC	CTP-V-Tan AUC	AUC difference	95% CI	*P*
Overall cohort (*n* = 104 events)	0.811	0.856	0.045	−0.005 to 0.096	0.079
Mechanical thrombectomy (*n* = 43 events)	0.684	0.833	0.149	0.048 to 0.251	0.004
Intravenous thrombolysis (*n* = 61 events)	0.856	0.852	−0.004	−0.061 to 0.054	0.901

AUC differences were calculated as CTP-V-Tan AUC minus cCTA-Tan AUC. AUCs were compared using the DeLong test. AUC, area under the curve; CI, confidence interval; cCTA-Tan, conventional CTA Tan score; CTP-V-Tan, venous-phase CTP-derived CTA Tan-based score.

### 3.7 Exploratory Heatmap analysis

**Figure 6:**
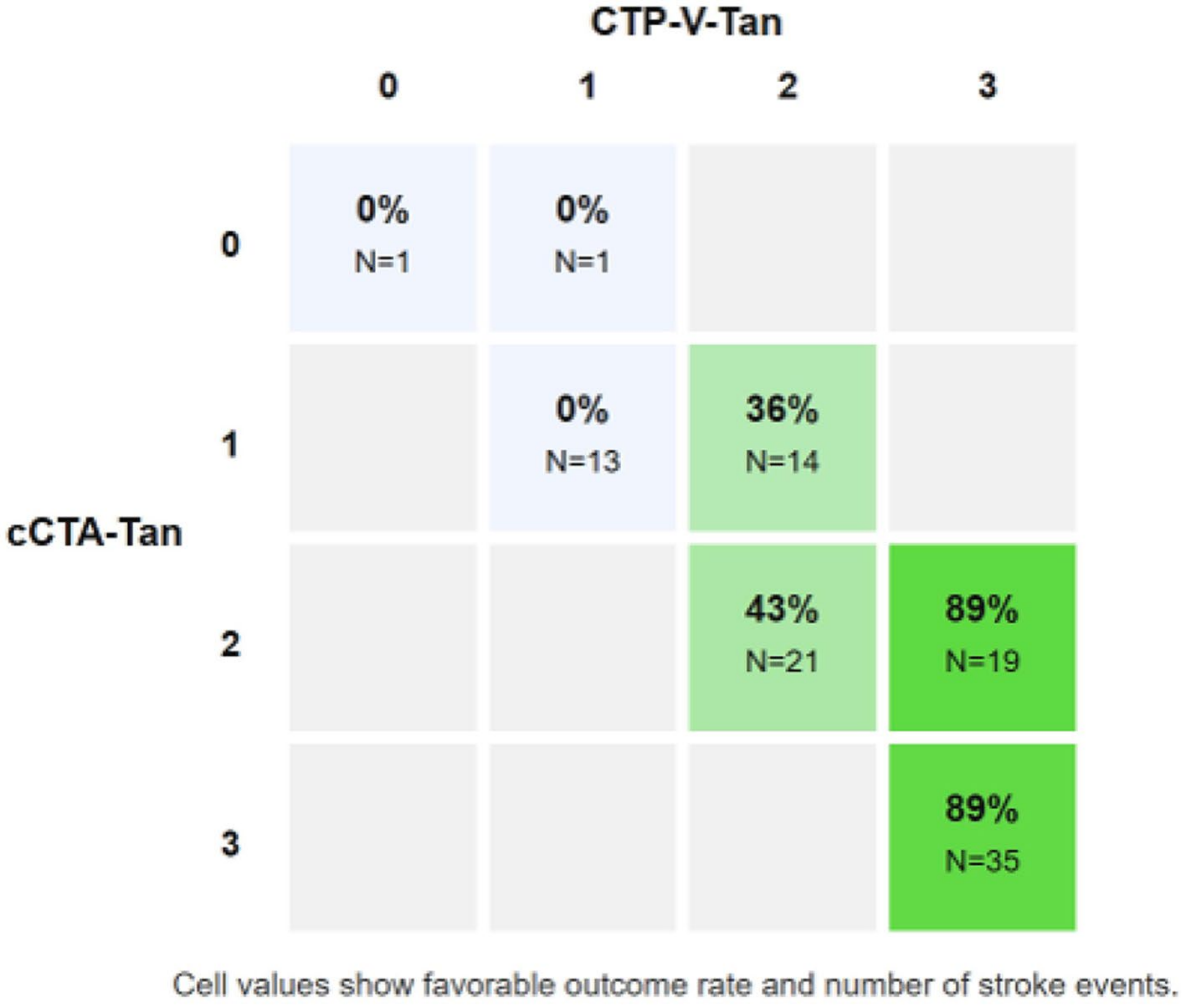
Exploratory heatmap of 90-day favorable functional outcome rates according to combinations of cCTA-Tan and CTP-V-Tan scores. Each cell shows the proportion of stroke events with a favorable functional outcome and the corresponding number of stroke events. Among stroke events with a cCTA-Tan score of 2, the favorable outcome rate was 43% for those with a CTP-V-Tan score of 2 and 89% for those with a CTP-V-Tan score of 3. Gray cells indicate score combinations with no observations. This analysis was exploratory and was not intended for formal hypothesis testing. Because several score combinations contained few observations, the cell-specific proportions should be interpreted cautiously.

In this exploratory analysis, the heatmap showed a gradient in 90-day favorable outcome rates across different combinations of cCTA-Tan and CTP-V-Tan. Among stroke events with a cCTA-Tan score of 2, the favorable outcome rate was 43% for those with a CTP-V-Tan score of 2 and 89% for those with a CTP-V-Tan score of 3. Among stroke events with a cCTA-Tan score of 1, the corresponding rates were 0% for a CTP-V-Tan score of 1 and 36% for a CTP-V-Tan score of 2. These observed patterns warrant further evaluation in larger cohorts. Because several score combinations contained few observations, the findings should be interpreted as descriptive and hypothesis-generating. The heatmap is shown in Figure 6.

### 3.8 Descriptive post-treatment radiological hemorrhagic transformation within 48 hours after treatment

As a descriptive post-treatment imaging finding, data on radiological hemorrhagic transformation within 48 h after treatment were available for 95 of the 104 stroke events; the remaining nine stroke events lacked follow-up imaging within this time window. According to the ECASS I imaging criteria, hemorrhagic transformation occurred in 36 of the 95 stroke events. The rate was 24.6% (14/57) in the favorable outcome group and 57.9% (22/38) in the unfavorable outcome group (*p* = 0.001).

## 4 Discussion

This study compared conventional single-phase CTA Tan scores with time-specific Tan-based collateral scores on multiphase CTP-derived CTA for discriminating 90-day functional outcomes. Four main findings emerged. First, CTP-V-Tan showed the highest AUC in the overall cohort (0.856 vs. 0.811 for cCTA-Tan), although the difference did not reach statistical significance in the DeLong test (*p* = 0.079). Second, CTP-V-Tan remained associated with 90-day favorable functional outcome after adjustment for measured clinical and imaging covariates. Third, CTP-V-Tan showed a higher AUC than cCTA-Tan in the mechanical thrombectomy subgroup, whereas no significant difference was observed in the intravenous thrombolysis subgroup; however, the treatment interaction was not statistically significant. Fourth, the exploratory heatmap showed differences in observed outcome proportions across score combinations, particularly among patients with intermediate cCTA-Tan scores. Because several cells contained few observations, these patterns were considered descriptive and hypothesis-generating. Taken together, the findings suggest that CTP-V-Tan may reflect delayed collateral filling and complement conventional single-phase CTA assessment, but the subgroup pattern should be considered exploratory.

Importantly, this study did not propose a new collateral scoring system. Instead, the Tan criteria were applied to different phases of CTP-derived CTA to obtain time-specific collateral scores. These scores should be considered a temporal extension of conventional CTA assessment rather than a replacement for the original Tan scoring system. In the overall cohort, the AUC of cCTA-Tan was 0.811, which was higher than the value of approximately 0.60–0.61 reported for the Tan collateral score alone in predicting 3-month functional independence in the HERMES collaboration, which pooled data from multiple randomized trials of endovascular treatment for anterior circulation large-vessel occlusion (10). However, after stratification by treatment, the AUC of cCTA-Tan was 0.684 in the mechanical thrombectomy subgroup, closer to that reported by HERMES. The higher AUC in the overall cohort was largely influenced by the greater discriminative performance observed in the intravenous thrombolysis subgroup. Therefore, our findings should not be interpreted as indicating that cCTA-Tan consistently achieves a high AUC across all stroke populations. They should instead be interpreted in the context of the single-center sample, case mix, distribution of treatment modalities, and dichotomization of functional outcome.

The high correlation between cCTA-Tan and CTP-V-Tan, the VIF results, the wide confidence interval for CTP-V-Tan, and the marked change in the cCTA-Tan estimate in the combined model indicate substantial shared information and limited precision. Accordingly, the individual coefficients in the combined model should be interpreted cautiously and not as evidence of independent prognostic contributions or statistical superiority. Nevertheless, CTP-V-Tan may reflect overlapping collateral information while also capturing later-phase patterns of delayed collateral filling. The higher AUC of CTP-V-Tan than cCTA-Tan observed within the mechanical thrombectomy subgroup should be considered hypothesis-generating because the formal treatment interaction was not significant. By contrast, no significant difference was observed in the intravenous thrombolysis subgroup, possibly because of greater heterogeneity in occlusion patterns and lesion burden, as well as the greater influence of thrombus burden and treatment timing (21).

Several collateral scoring systems have been developed for different CTA acquisition strategies. The Tan score is a simple collateral score originally used on single-phase CTA, whereas the Menon multiphase CTA (mCTA) collateral score was specifically designed for multiphase CTA and incorporates delayed collateral filling across sequential phases. Previous studies have reported inconsistent findings regarding the added prognostic value of multiphase CTA. Menon et al. showed that mCTA provided time-resolved assessment of pial arterial filling and improved outcome prediction compared with single-phase CTA and perfusion CT models (22). By contrast, Schregel et al. reported that collateral status assessed on single-phase CTA was sufficient for outcome prediction in their optimized single-phase CTA protocol (23). These differences suggest that the relative value of single-phase and multiphase collateral assessment may depend on acquisition protocol, scan timing, scoring system, and patient selection. In the present study, we did not use the Menon mCTA collateral score because our aim was not to compare different collateral grading systems. Instead, we applied the same Tan-based framework to conventional CTA and to different phases of CTP-derived CTA to examine whether temporal information from CTP source images provides additional prognostic information. This design reduced the confounding that would occur if both the imaging phase and the scoring system were changed simultaneously. Arterial-phase CTP-derived CTA did not outperform conventional CTA, whereas venous-phase CTP-derived CTA showed the highest AUC. This pattern suggests that delayed collateral filling visible on later CTP-derived CTA phases may provide complementary information about collateral status. However, early arterial-phase images may underestimate delayed collaterals, whereas late venous-phase images may overestimate collateral filling. Therefore, CTP-V-Tan should be interpreted as a Tan-based time-specific collateral score rather than as a replacement for the original Tan score or established mCTA scores. Previous CTP studies also support the clinical value of dynamic imaging information. CTP-derived collateral indices may outperform single-phase CTA in identifying favorable reperfusion or collateral status (24, 25). Perfusion-derived parameters, including relative cerebral blood flow and the hypoperfusion intensity ratio, have been associated with CTA-defined collateral status (26, 27), as well as functional outcome, infarct volume, and malignant cerebral edema after mechanical thrombectomy (28, 29). Radiological hemorrhagic transformation within 48 h after treatment was more frequent in patients with an unfavorable outcome; however, functional outcome is multifactorial and cannot be explained by hemorrhagic transformation alone.

This study has several limitations. First, it was a single-center retrospective study with a limited sample size. The study was not powered to establish small AUC differences or treatment-specific effects; therefore, the thrombectomy-subgroup finding requires validation in larger multicenter cohorts. In addition, because eligibility required complete pretreatment non-contrast CT, conventional CTA, CTP, reconstructable CTP-derived CTA, and 90-day mRS data, the cohort may not fully represent all reperfusion-treated AIS patients. Treatment modality was modeled as mechanical thrombectomy versus intravenous thrombolysis without subsequent thrombectomy, which may not fully account for heterogeneity between direct thrombectomy and bridging therapy. Premorbid mRS was not consistently available, which may have introduced outcome misclassification or residual confounding in patients with prestroke disability. Second, although thrombectomy-related variables were explored in sensitivity analyses, the limited number of thrombectomy events did not allow all important prognostic factors to be entered simultaneously into one stable model. Third, although no reperfusion treatment was initiated during the interval between CTP and conventional CTA, the absolute timing difference between CTP-derived CTA and conventional CTA may still have had a minor influence on collateral appearance. The quality of CTP-derived CTA images may also be affected by acquisition protocols, post-processing methods, patient motion, and cardiac function. Their reproducibility across centers remains to be established. Although all included cases had analyzable time-attenuation curves and reconstructable arterial, arteriovenous, and venous phases within the 45-s CTP acquisition window, markedly delayed collateral filling beyond this window could not be captured or analyzed. Fourth, venous-phase images may be affected by enhancement of cortical veins, venous sinuses, and draining veins, potentially leading to overestimation of delayed arterial-like collateral filling during CTP-V-Tan assessment. Because earlier-phase images were reviewed before CTP-V-Tan scoring, venous-phase assessment was not fully independent of preceding phases and may be susceptible to phase-order anchoring bias. To reduce this bias, the culprit vessel and affected vascular territory were identified using earlier-phase images, typical venous structures were excluded, and images were evaluated by two radiologists with interobserver agreement analysis. Furthermore, the venous phase was restricted to images acquired near the peak of the venous output curve. Persistent post-peak enhancement, delayed venous washout, and impaired venous clearance were not quantitatively assessed. These post-peak temporal features may have independent prognostic value, but their imaging significance differs from that of CTP-V-Tan evaluated in this study. They should be investigated separately as arteriovenous temporal parameters. The lower interobserver agreement for CTP-V-Tan compared with cCTA-Tan probably reflects the greater difficulty of distinguishing delayed arterial-like collateral filling from venous structures on delayed-phase images. Therefore, clinical application of CTP-V-Tan requires standardized phase selection and combined review of maximum-intensity projection images, thin-section source images, and multiplanar reconstructions. Further standardization using image registration, dynamic 4D-CTA visualization, or automated vessel segmentation is warranted. One patient contributed two temporally distinct stroke events, which may have introduced minimal within-patient dependence. The cohort also included a small number of ACA occlusions and thrombolysis-treated anterior-circulation events without visible major vessel occlusion. Although these cases reflect real-world reperfusion-treated anterior-circulation stroke practice, Tan-based collateral grading is less anatomically standardized in these presentations than in ICA/MCA occlusion. Therefore, the ICA/M1 and ICA/M1/M2 sensitivity analyses should be interpreted as supportive analyses, and external validation in anatomically more homogeneous cohorts remains necessary. Finally, quantitative CBF, CBV, and MTT values were not evaluated as primary predictors because they were not consistently available as standardized patient-level variables in this retrospective dataset, and the study question focused on whether CTP source-image-derived collateral information could supplement conventional CTA Tan scoring. Future studies should compare CTP-derived Tan-based collateral scores with quantitative perfusion parameters and automated collateral indices in larger cohorts.

## 5 Conclusion

CTP-V-Tan provides a phase-specific assessment of delayed collateral filling and was associated with 90-day favorable outcome after reperfusion therapy in patients with acute anterior circulation ischemic stroke. Although its AUC was numerically higher than that of cCTA-Tan, the difference was not statistically significant in the overall cohort. A higher AUC for CTP-V-Tan was observed within the mechanical thrombectomy subgroup, but the treatment interaction was not significant, and this exploratory finding requires external validation. This approach uses routinely acquired CTP source images without requiring additional scanning and may complement conventional CTA collateral assessment. Larger multicenter studies are needed to validate its reproducibility and clinical utility.

## Data Availability

The datasets analyzed in this study are not publicly available because they contain potentially identifiable clinical and imaging information and are subject to patient privacy and institutional ethical restrictions, further inquiries can be directed to the corresponding author/s.

## Glossary

AIS: Acute ischemic stroke
CTA: Computed tomography angiography
CTP: Computed tomography perfusion
CT: Computed tomography
mRS: modified Rankin Scale
NIHSS: National Institutes of Health Stroke Scale
ASPECTS: Alberta Stroke Program Early CT Score
ROI: Region of interest
TAC: Time–attenuation curve
ROC: Receiver operating characteristic
AUC: Area under the curve
OR: Odds ratio
CI: Confidence interval
MCA: Middle cerebral artery
ACA: Anterior cerebral artery
ICA: Internal carotid artery
mTICI: modified Thrombolysis in Cerebral Infarction
CBF: Cerebral blood flow
CBV: Cerebral blood volume
MTT: Mean transit time
VIF: Variance inflation factor
ECASS: European Cooperative Acute Stroke Study
cCTA-Tan: Conventional CTA Tan score
CTP-A-Tan: Arterial-phase CTP-derived CTA Tan-based score
CTP-AV-Tan: Arteriovenous-phase CTP-derived CTA Tan-based score
CTP-V-Tan: Venous-phase CTP-derived CTA Tan-based score
mCTA: multiphase CTA
HERMES: Highly Effective Reperfusion Evaluated in Multiple Endovascular Stroke Trials
